# Bone metastases from renal cell carcinoma: patient survival after surgical treatment

**DOI:** 10.1186/1471-2474-11-145

**Published:** 2010-07-03

**Authors:** Andreas Fottner, Melinda Szalantzy, Lilly Wirthmann, Michael Stähler, Andrea Baur-Melnyk, Volkmar Jansson, Hans Roland Dürr

**Affiliations:** 1 Department of Orthopaedic Surgery, Campus Grosshadern, Ludwig-Maximilians-University Munich, Marchioninistr 15, 81377 Munich, Germany; 2 Department of Urology, Campus Grosshadern, Ludwig-Maximilians-University Munich, Marchioninistr 15, 81377 Munich, Germany; 3 Department of Radiology, Campus Grosshadern, Ludwig-Maximilians-University Munich, Marchioninistr 15, 81377 Munich, Germany

## Abstract

**Background:**

Surgery is the primary treatment of skeletal metastases from renal cell carcinoma, because radiation and chemotherapy frequently are not effecting the survival. We therefore explored factors potentially affecting the survival of patients after surgical treatment.

**Methods:**

We retrospectively reviewed 101 patients operatively treated for skeletal metastases of renal cell carcinoma between 1980 and 2005. Overall survival was calculated using the Kaplan-Meier method. The effects of different variables were evaluated using a log-rank test.

**Results:**

27 patients had a solitary bone metastasis, 20 patients multiple bone metastases and 54 patients had concomitant visceral metastases. The overall survival was 58% at 1 year, 37% at 2 years and 12% at 5 years. Patients with solitary bone metastases had a better survival (p < 0.001) compared to patients with multiple metastases. Age younger than 65 years (p = 0.036), absence of pathologic fractures (p < 0.001) and tumor-free resection margins (p = 0.028) predicted higher survival. Gender, location of metastases, time between diagnosis of renal cell carcinoma and treatment of metastatic disease, incidence of local recurrence, radiation and chemotherapy did not influence survival.

**Conclusions:**

The data suggest that patients with a solitary metastasis or a limited number of resectable metastases are candidates for wide resections. As radiation and chemotherapy are ineffective in most patients, surgery is a better option to achieve local tumor control and increase the survival.

## Background

Renal cell cancer (RCC) is among the 10 most common cancers in both men and women. It comprises 2-3% of all malignancies [1]. The incidence of RCC in the United States has been increasing at a rate of approximately 3% per year [2]. Approximately one third of patients with newly diagnosed RCC have metastatic disease at the initial presentation [3]. The most common site for metastasis from RCC is the lung (50% of patients), followed by the skeleton (20% to 50% of patients) [4,5].

Approximately 50% of patients presenting with metastases die within the first year and only 10% survive more than 5 years [6]. Compared to other types of carcinoma frequently affecting bone the prognosis of RCC is better than for lung cancer, but not as good as for breast or prostate cancer [2]. The main reason for the poor prognosis is the poor response of RCC metastases to radiation and chemotherapeutic regimens [7].

Patients with solitary bone metastasis from RCC reportedly have the best prognosis, with a 5 year-survival rate between 35% and 60% [8]. Owing to the longer survival of patients with solitary bone metastasis, a number of authors recommend a surgical approach with curative intent and implant stabilization to prevent local disease progression [7-11].

The aim of this study was to identify clinical, pathological and surgical factors that are associated with better survival of patients after surgical treatment of skeletal metastases from RCC.

## Methods

We retrospectively reviewed a consecutive series of 101 patients surgically treated for osseous metastases from RCC between 1980 and 2005. Clinical data for these patients were obtained through hospital records and the patient database system. The minimum clinical follow up was 24 months or until death (range 1-244 months). Due to the clear retrospective nature of the study an approval by the ethic committee was not necessary at our university. Perioperative variables collected were age and gender of the patient, type of surgery, complications, time period after first diagnosis of RCC and after onset of symptoms, metastatic pattern, location of metastases, incidence of pathologic fracture and local recurrence and time of survival.

Preoperative investigations included conventional radiographs, CT scan or MRI to evaluate the size and site of the tumor. If the diagnosis of metastasis was in question, a preoperative needle biopsy was obtained to confirm the histological diagnosis. CT scans of the chest, abdomen and pelvis were performed to evaluate visceral metastases. In all cases the diagnosis of metastatic RCC was confirmed by the intraoperative obtained histological examination.

The surgical treatment for bone metastases varied in each patient. Factors influencing the choice of surgery included age, disease status, symptoms, morbidity of the patient, the location and extend of bone disease, existence of extraosseous metastases and the patient's wishes. Until the year 1997 we pursued a comparatively palliative treatment concept with limited surgery. After that time as a result of reports in the literature, we changed to a surgical approach with more curative intent, especially for patients with localized metastatic disease [9,11-14]. This included an interdisciplinary surgical regimen for patients with a limited amount of additional visceral metastases. This subpopulation was also analysed.

Survival was analyzed with respect to location and dissemination of metastases, age, interval after diagnosis of RCC, existence of pathologic fractures, local recurrences, margin of the resection and the use of chemotherapy and radiation. Results of the palliative surgical treatment used until 1997 were compared with those surgical approach with more curative intent used after 1997.

Statistical analyses were performed using MedCalc v 10.3.1 (MedCalc Software, Mariakerke, Belgium). Incidences of local recurrence in respect to different treatment regimes were analysed using the McNemar test. The survival distribution for overall survival was estimated using the Kaplan-Meier method [15]. Correlations of survival were calculated using the log-rank test with *p *< 0.05 defined as significant.

## Results

A total of 116 surgical procedures were performed in 101 patients. The demographic data of the patients are shown in Table 1. 26.7% had solitary bone metastases, 19.8% had multiple bone metastases and 53.5% had concomitant visceral metastases at the time of presentation. In 33% of patients osseous metastases were detected at the same time as the RCC was diagnosed.

**Table 1:** Demographics

Gender	
Male	71
Female	30
Age at time of surgery	
Median	64.5 years
Interquartile range	57.3-72.1 years
Time between diagnosis of RCC and osseous metastasis	
Median	9.7 months
Interquartile range	1.0-49.0 months
Sites	116
Spine	29
Upper extremity	40
Clavicle/scapula	6
Humerus	28
Forearm	5
Lower extremity	47
Pelvis	15
Femur	29
Tibia	3
Foot	1
Symptoms at time of presentation	
Pain	83%
Pathologic fracture	36%
Neurological deficit	11%
Tumor	20%
Duration of symptoms before presentation	
Median	2.0 months
Interquartile range	1.0-4.3 months

According to the Enneking classification [16] 8 (6.9%) radical resections, 18 (15.5%) wide excisions, 64 (55.2%) marginal excisions and 26 (22.4%) intracapsular excisions were performed. The types of surgery performed are shown in table 2. Four patients (3.4%) died within a postoperative period of 30 days. The complication rate after surgery was 9.5% (11/116). The type and quantity of complications is shown in table 3. We included those complications that either prolonged the hospitalisation or made a second surgical intervention necessary. For 78 patients (77.2%) the treatment included radiation, 19 patients before and 59 patients after the surgical procedure. Chemotherapy was used in 36 cases (35.6%) (19 patients (18.8%) preoperatively and 17 patients (16.8%) postoperatively).

**Table 2:** Types of Surgery

Radical resection/amputation	8
Wide excision	18
Resection arthroplasty	14
Structural graft	4
Marginal excision	64
Resection arthroplasty	16
Plate fixation	33
Intramedullary nail	4
Without internal fixation	11
Intracapsular excision	26
Resection arthroplasty	7
Plate fixation	6
Intramedullary nail	5
Without internal fixation	8

**Table 3:** Complications

Wound healing disorder	2
Nerve palsy	3
Haematoma (revised)	1
Infection of endoprosthesis	1
Dislocation of endoprosthesis	1
Pneumonia	2
Fracture	1

17 patients (16.8%) developed a local recurrence. 11 of these 17 cases (64.7%) were treated by reresection of the tumor. Two patients with local recurrence received radiation therapy for pain reduction, and 4 patients were not treated owing to tumor progression. Radiation did significantly (p = 0.377) not affect the incidence of local recurrence. A local recurrence occurred in 10.5% (2/19) of patients who received radiation treatment preoperatively, in 18.6% (11/59) of patients who received radiation treatment postoperatively, and in 17.4% (4/23) of patients who did not receive radiation treatment. Local recurrence was significantly (p < 0.001) less frequent in patients with negative resection margins 4.7% (1/21), when compared to those with tumor-infiltrated margins 20% (16/80).

**Figure 1:**
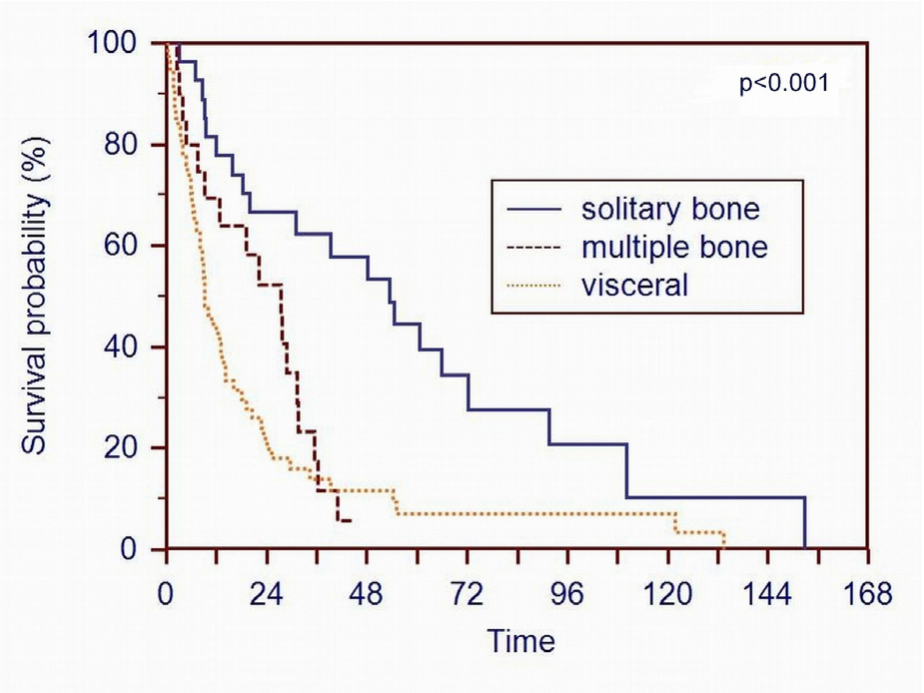
**A Kaplan-Meier survival curve of patients based on the metastatic pattern shows that patients with solitary bone metastases had a better survival (p < 0.001) than patients with multiple bone metastases or additional visceral metastases**.

The overall survival rates of all 101 patients were 58.4% at 1 year, 36.6% at 2 years, 23.8% at 3 years and 11.9% at 5 years. The mean survival time was 26.9 months, median survival 15.8 months (interquartile range: 6.8 - 34.6 months). The difference in survival between patients with solitary bone metastases, multiple bone metastases, and additional visceral metastases was statistically significant (p < 0.001) (Figure 1). Patients with a solitary bone metastasis had a significantly better (p = 0.002) survival than patients with multiple bone metastases.

**Figure 2:**
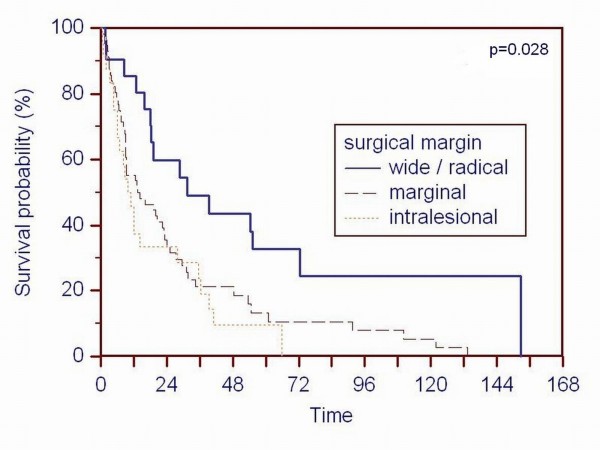
**A Kaplan-Meier survival curve of patients based on the surgical margin according to the Enneking classification **[16]**shows that patients with a tumor-free resection margin have a better survival rat (p = 0.028) than patients with a tumor-infiltrated resection margin**.

**Figure 3:**
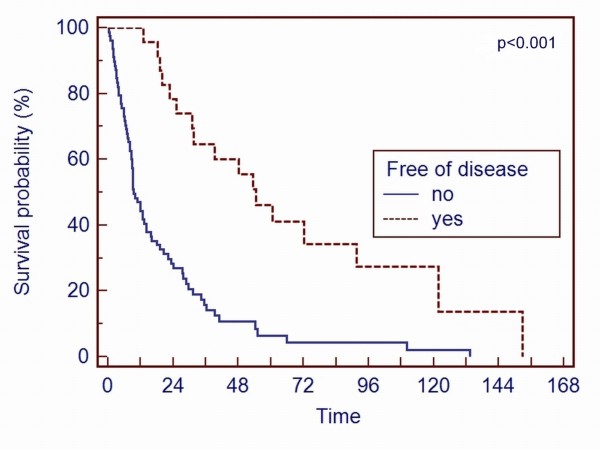
**A Kaplan-Meier survival curve of patients stratified on the basis of being diagnosed as "free of disease" or not shows that patients with the diagnosis "free of disease" at any time after the operation had a better survival (p < 0.001)**.

The histologic resection margin also affected overall survival. Patients with a tumor-free resection margin had a significant better (p = 0.028) survival (Figure 2). Patients diagnosed as being free of disease any time after the operation had the best overall survival (p < 0.001) (Figure 3). 16 of the these 23 patients had additional visceral metastases resected in an interdisciplinary regimen to achieve the status of "free of disease". 13 resections were performed before and 3 resections after the surgical intervention of the bone metastasis. This subpopulation had also a better (p < 0.001) survival.

**Figure 4:**
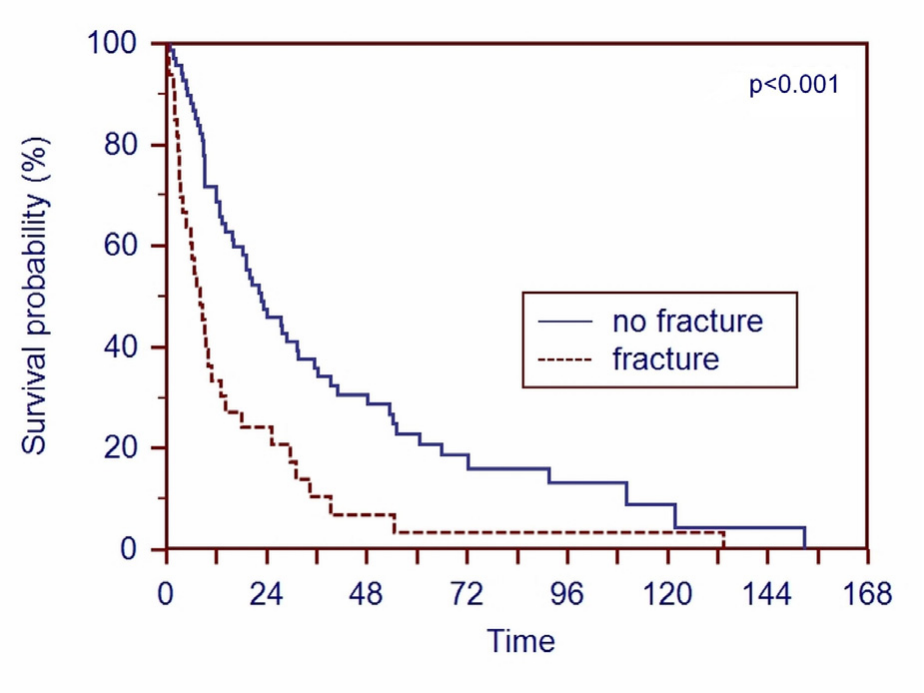
**A Kaplan-Meier survival curve of patients based on whether a pathologic fracture was present before the operation or not shows that presence of a pathologic fracture decreases the prognosis of the patient (p < 0.001)**.

Patients with a pathologic fracture had a significantly shorter (p < 0.001) survival compared with patients without pathologic fracture (Figure 4).

Of the variables analyzed: gender, time after diagnosis of RCC, localization of the metastases, incidence of local recurrence and the use of chemotherapy or radiation before or after surgery did not have a significant effect on the overall survival (Table 4). The alteration of the surgical approach in 1997 to a more curative treatment concept also had no influence (p = 0.67) on the overall survival. Only patients with solitary bone metastases had a better (p = 0.048) survival with the more curative approach.

**Table 4:** Analysis of overall survival

Variable/Subgroup	Number of patients	Overall survival at 1 year	Overall survival at 5 years	p-Value
Metastatic pattern				< 0.001
Solitary bone	27	0.78	0.44	
Multiple bone	20	0.69	0.10	
Bone and visceral	54	0.43	0.06	
Local recurrence				0.060
yes	17	0.82	0.14	
no	84	0.52	0.12	
Gender				0.765
Female	30	0.52	0.19	
Male	71	0.59	0.16	
Age				0.036
< 65 years	53	0.62	0.24	
> 65 years	48	0.52	0.07	
Time of operation				0.670
After 1997	49	0.58	0.18	
Before 1997	52	0.56	0.15	
Time after diagnosis of RCC				0.081
< 2 years	64	0.48	0.14	
> 2 years	37	0.72	0.19	
Localisation				0.817
Appendicular	58	0.56	0.15	
Axis	43	0.58	0.15	
Adjuvant chemotherapy				0.370
None	65	0.55	0.13	
Pre-operative	19	0.56	0.14	
Post-operative	17	0.65	0.30	
Radiation				0.861
None	23	0.61	0.16	
Pre-operative	19	0.62	0.17	
Post-operative	59	0.54	0.16	

## Discussion

We were trying to identify factors that are associated with better survival of patients after surgical treatment of skeletal metastases from RCC. Patients with osseous metastases of RCC have an unfavorable prognosis. In some studies, more than 50% of patients die within the first year [7,9,10,17,18]. The survival rate for our patients was with 58.4% at 1 year and 11.9% at 5 years slightly better. However, in some studies patients had a better survival rate [8,12,19], but Althausen et al. 1997 [12] and Tobisu et al. 1989 [19] studied small patient sample, and Fuchs et al. 2005 [8] investigated only patients with solitary bone metastases.

Comparable to other studies, the Kaplan-Meier survival rate curves (Figure 1, 2, 3 and 4) contain two main features: survival decreases steeply in the first year, and the curve diminishes after a period of proximally 24 months.

The first, and most important, decision of a surgeon dealing with patients who have metastases from RCC is to select the appropriate treatment concept with respect to the extent of the metastatic disease and prognosis of the patient. For patients with the potential of becoming a long-term survivor, an aggressive surgical resection should be considered [7,10,11].

The metastatic pattern with solitary bone metastases has been identified as having the strongest association with survival [7-11,20,21]. We came to the same conclusion; patients with solitary bone metastases had a better survival rate than patients with multiple bone metastases or visceral involvement.

We found patients younger than 65 years and patients without a pathologic fracture at the time of surgery have a better prognosis. Two other studies [8,17] came to the same conclusions concerning younger patients. But most studies examining patients with metastases from RCC have reported no correlation concerning the age with survival [7,9,10,12,20,21]. The influence of pathologic fractures on survival was analyzed in a few studies. Although some studies [20,22-24] verified that patients with a pathologic fracture had an unfavorable survival, one study could not support the importance of pathologic fractures [12].

Factors that did not influence survival in our study were gender, local recurrence, radiation, chemotherapy and location of metastases. Although only one study associated male gender with prolonged survival [8], gender was not important in other studies [4,7,9,10,12,17,20]. The influence of local recurrence on survival was analyzed in only one study [20]. This study confirms our suggests that a local recurrence of the tumor does not decrease the prognosis.

A controversial factor is the influence of the location of metastases on the prognosis. Although two studies [7,12] show there is a difference in the survival rate between metastases of the axial compared with the appendicular skeleton, our findings, and two other studies [4,7] could not detect an influence of the location.

For patients with multiple metastases and a limited survival time, surgery remains the preferred therapy because RCC often is resistant to chemotherapy and radiation therapy [8,10,12,24-27]. Even though radiation of osseous metastases can be useful for pain control and prevention of fractures [28], surgery is more effective in restoring function and preventing local tumor progression [11,24]. The analysis of the 17 cases of local recurrence in our patients did not show a preventive effect of radiation for local tumor progression. On the contrary, the incidence of local recurrence was less frequent for surgical procedures with tumor-free margin.

As bone metastasis stands for an advanced stage of disease, it was considered a secondary goal to achieve cure of disease by radical resection of the tumor. However, favorable survival rates after successful radical resection of bony metastases justify a surgical approach with more curative intent [7-10,20,29,30]. The therapeutic concept at our institution was changed in 1997 to a more curative approach. We believe this was the right decision, as we observed a substantial improvement in survival with the new therapeutic concept for patients with solitary metastasis.

Regarding the operative procedures, our data confirm other studies that a resection with a tumor-free margin increases the survival rate [7,8,12,24]. We believe one should aim for the widest resection of metastases as technically possible. Even patients with a combination of resecable osseous and visceral metastases are candidates for an interdisciplinary curative surgical regimen because the status of "free of disease" gave the patients in our study the best chance to become long-term survivors (> 40% survival rate after 5 years).

## Conclusions

Similar to other studies we found that patients with solitary metastases without pathologic fracture have the best prognosis for long-term survival. More controversial predictors are age, gender, local recurrence, the interval after diagnosis of RCC and the location of the metastases. For patients with mid-term to long-term prognosis, surgeons should try to achieve tumor-free margins to enhance the survival rate and achieve local tumor control. This also applies to patients with a limited amount of resectable osseous and visceral metastases. As radiation and chemotherapy have little effect on survival, even patients with an unfavorable prognosis should be considered for surgery.

## Competing interests

The authors declare that they have no competing interests.

## Funding

No benefits or funds were received in support for the study.

## Authors' contributions

AF: preparation of the manuscript, statistical analysis; MS: data collection; LW: preparation of the manuscript; MS: data collection; AB: data collection; VJ: revision of the manuscript; HRD: study design, revision of the manuscript. All authors read and approved the manuscript.

## Pre-publication history

The pre-publication history for this paper can be accessed here:

http://www.biomedcentral.com/1471-2474/11/145/prepub
